# Unveiling the Unusual: A Unique Case of Verrucous Cyst

**DOI:** 10.7759/cureus.53343

**Published:** 2024-01-31

**Authors:** Zaryab Alam, Michelle S Bach, Mojahed Shalabi, Shannon C Brown

**Affiliations:** 1 Medicine, Texas A&M College of Medicine, Bryan, USA; 2 Medicine, University of Texas at Austin Dell Medical School, Austin, USA; 3 Internal Medicine, Baylor Scott & White All Saints Medical Center, Fort Worth, USA; 4 Dermatology, Baylor Scott & White Health, Temple, USA

**Keywords:** cytological atypia, pcr test, epidermoid cysts, human papillomavirus (hpv), verrucous cysts

## Abstract

Verrucous cysts are uncommon types that cannot be distinguished from epidermal inclusion cysts clinically and require histopathological analysis and human papillomavirus (HPV) polymerase chain reaction (PCR) for accurate diagnosis. The pathogenesis of verrucous cysts is thought to involve HPV infection, either of an existing cyst or through direct infection of keratinocytes, leading to new cyst formation. While verrucous cysts can affect individuals of any sex and are typically found on the trunk, extremities, and face, they are particularly notable for their potential association with high-risk HPV types, such as 16 and 18, which may lead to malignant transformation.

In this report, we present the case of a 48-year-old female with a history of endometriosis and pelvic inflammatory disease, who sought evaluation for a persistent subcutaneous nodule on her right flank. The patient reported pain, a recent color change, and an increase in the nodule size. Clinical examination revealed a 2.7 cm subcutaneous nodule with a central brown-gray papule. Despite no history of dermatologic malignancies, the nodule was excised, and subsequent histopathological examination confirmed a diagnosis of a ruptured verrucous cyst. The cyst exhibited acanthotic papillomatous squamous epithelium without cytologic atypia and koilocytic change in cells.

This case offers direct and valuable insights into the clinical presentation, diagnosis, and management of verrucous cysts. It highlights the importance of a thorough diagnostic approach, combining histopathological examination with HPV PCR testing, to accurately differentiate verrucous cysts from other similar cutaneous lesions. The report also emphasizes the need for vigilance in managing these cysts due to their potential association with high-risk HPV types and the consequent risk of malignant transformation. These insights contribute significantly to the existing body of literature on verrucous cysts and aim to enhance clinical awareness and patient care in dermatology.

## Introduction

Verrucous cysts represent a unique and rare dermatological entity, characterized as non-plantar, human papillomavirus (HPV)-infected epidermoid cysts that exhibit distinct verrucous features on histopathological examination [1,2]. Unlike verruca vulgaris, which is commonly observed in pediatric populations, verrucous cysts predominantly occur in adults, presenting a different clinical challenge [3]. These cysts are notable for their equal prevalence across all sexes, and they typically do not connect with the surface of the epidermis [4,5]. They are most frequently located on the trunk, extremities, and face [4,5]. The unique presentation and location of these cysts, coupled with their potential association with HPV, make them a subject of clinical interest. Furthermore, the cosmetic and psychological impacts of these cysts, particularly in visible areas, add to the urgency of their proper diagnosis and management [4,5].

This case report details the presentation, diagnosis, and management of a verrucous cyst in an adult patient. In addition to the rarity of verrucous cysts, their potential to be associated with high-risk HPV types adds a layer of complexity to their management, particularly considering the risk of malignant transformation [6,7]. This report aims to enhance the understanding of verrucous cysts among clinicians and highlights the importance of considering them in the differential diagnosis of subcutaneous lesions in adult patients.

## Case presentation

**Figure 1:**
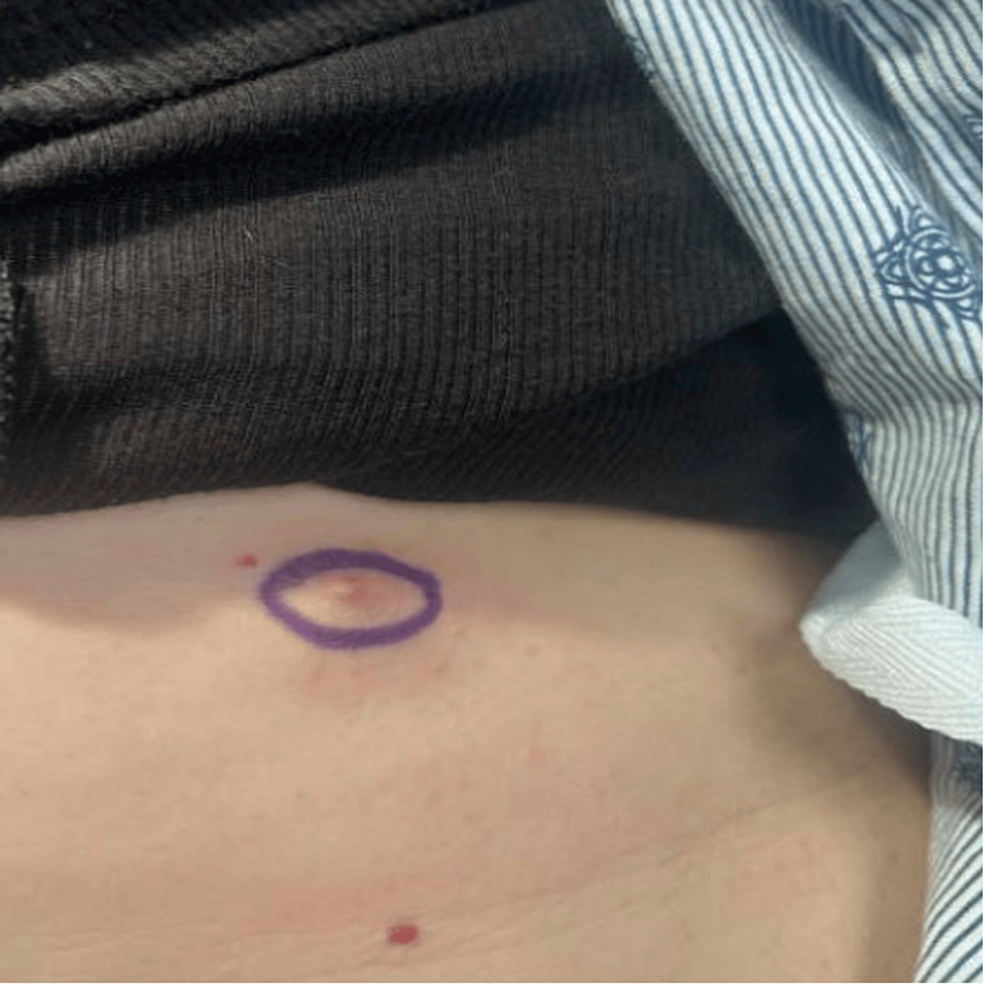
A 2.7 cm subcutaneous nodule with an overlying 3-mm brown-gray central papule

The patient, a 48-year-old female with a history of endometriosis and pelvic inflammatory disease, presented with a long-standing subcutaneous nodule on her right flank. The nodule, which had undergone changes in color and size, was causing the patient discomfort. Upon physical examination, a 2.7 cm subcutaneous nodule with a central 3-mm brown-gray papule was observed (Figure 1). The patient had no personal or family history of dermatologic malignancies, and prior pap smears were negative for high-risk HPV.

**Figure 2:**
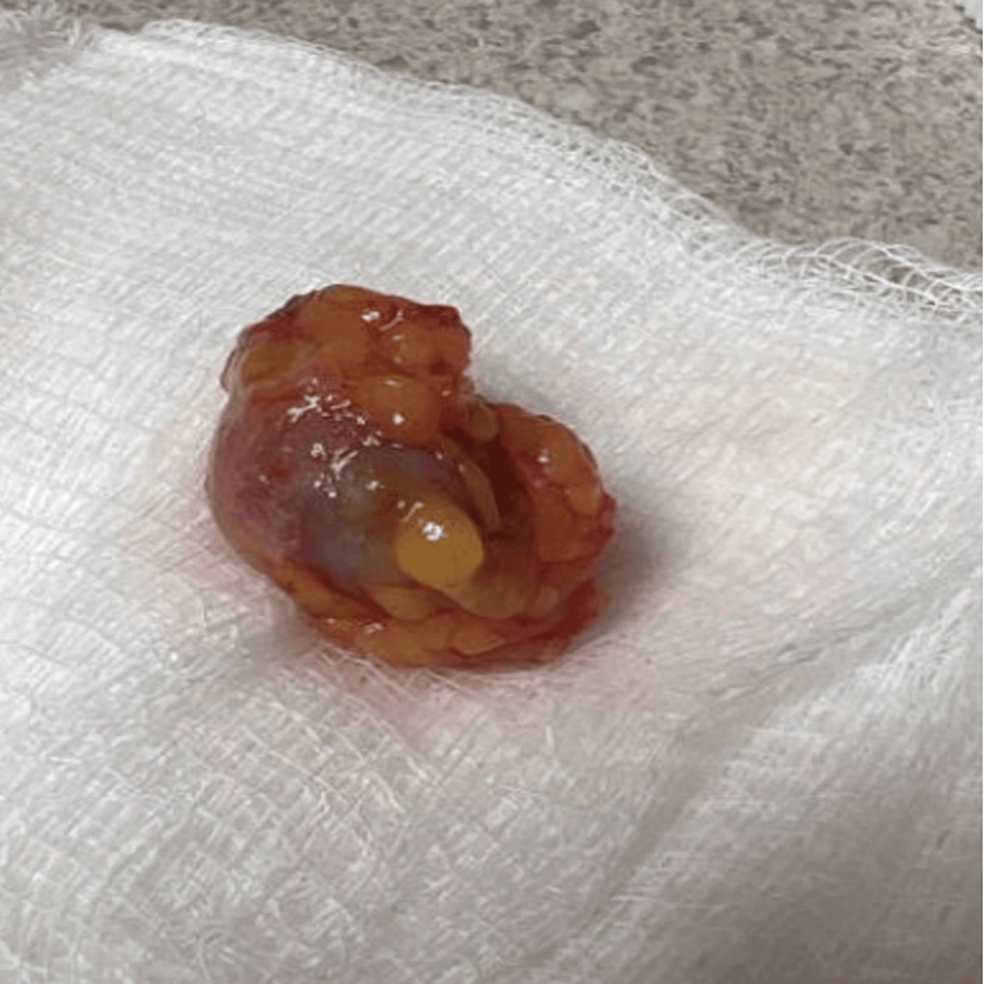
A 1.6 x 0.7 x 0.4 cm unoriented excision of tan-white skin with an attached 3.0 x 2.0 cm tan-pink portion with yellow, lobulated adipose

For the removal of the cyst, a local anesthetic was administered to ensure the patient's comfort during the procedure. The cyst was then surgically excised down to the underlying adipose tissue, ensuring complete removal (Figure 2).

**Figure 3:**
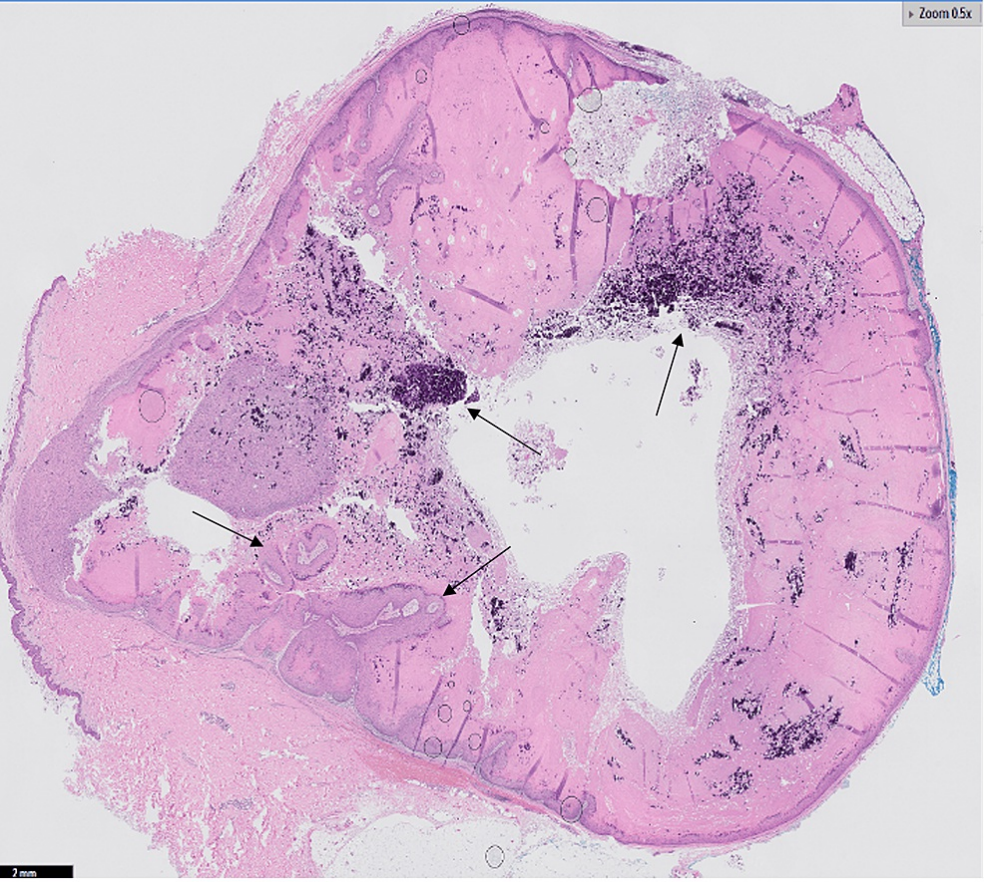
H&E demonstrated acanthotic papillated squamous epithelium with prominent hypergranulosis and whorls of keratinocytes without evidence of cytologic atypia consistent with ruptured verrucous cyst (black arrows)

**Figure 4:**
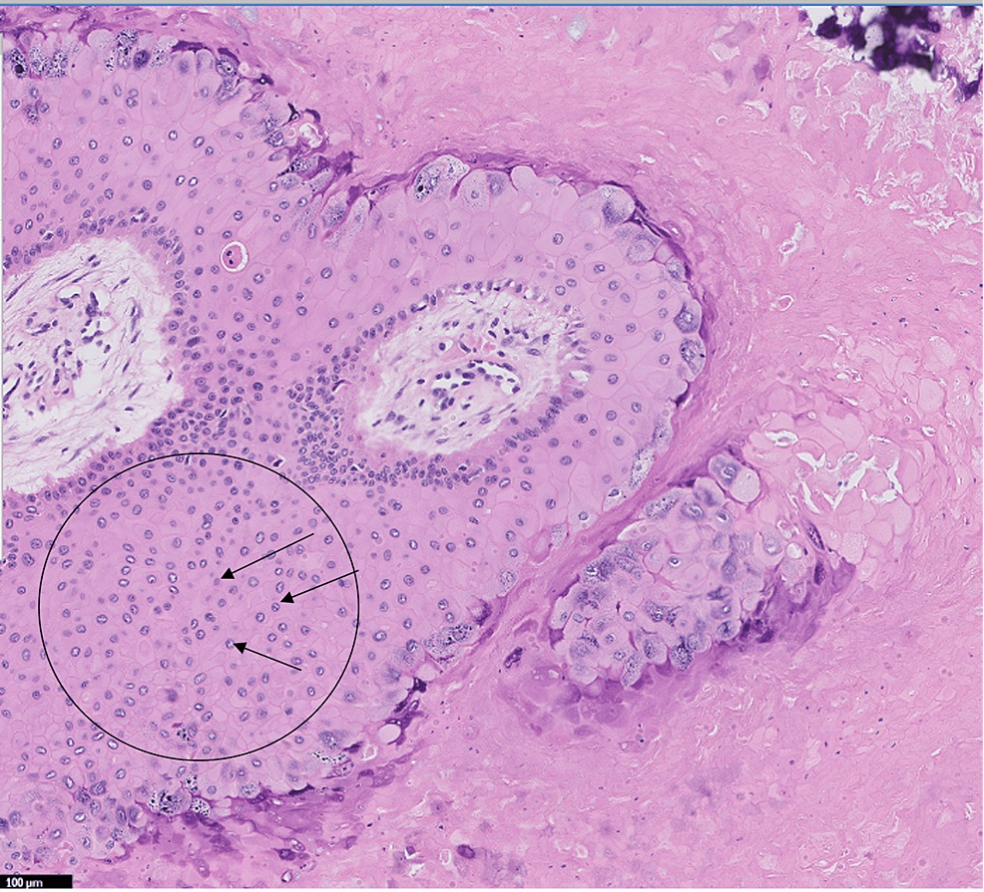
Higher magnification of koilocytic epithelial cells with characteristic hyperchromatic nuclei with perinuclear vacuoles (black arrows in black circle)

The excision was followed by an uncomplicated closure. Final histopathology revealed an acanthotic papillated squamous epithelium with prominent hypergranulosis and koilocytic epithelial cells, but without cytologic atypia, confirming the diagnosis of a ruptured verrucous cyst (Figures 3, 4).

The patient was informed of the diagnosis upon receiving the pathology report. She was offered further diagnostic workup with HPV polymerase chain reaction (PCR) but declined this option. The patient returned for suture removal two weeks post-surgery. To date, there have been no signs of recurrence. All relevant medications administered during the treatment and any complications that arose have already been mentioned in the report.

## Discussion

The evolving understanding of verrucous cysts since their initial description in 1991 has significantly increased [6,7]. Initially characterized by their histopathologic features without detectable HPV antigens, recent studies have established a connection between verrucous cysts and various HPV strains, including 59, 34, 24, 20, 16, 8, and 6, some of which are also associated with anogenital lesions [6,7]. This association has broadened the clinical perspective on these cysts, indicating a more complex etiology and potential risk factors than previously understood [6,7].

The rarity of verrucous cysts poses a challenge in its diagnosis [5,8]. However, based on the limited literature on this dermatological entity, it seems that they are an uncommon presentation with a distribution that does not appear to favor any specific demographic or geographic population [5,8]. Clinically, verrucous cysts can be mistaken for epidermal inclusion cysts, pilomatricomas, lipomas, and dermatofibromas due to their similar presentations [5,8]. This resemblance necessitates a combination of histopathological analysis and HPV PCR testing for accurate diagnosis [5,8]. Understanding the specific HPV strains involved is critical in assessing potential risks and guiding treatment decisions [5,9]. The pathogenesis of verrucous cysts remains an area of active research, with current hypotheses suggesting two potential pathways: HPV infection of an existing cyst or direct infection of keratinocytes leading to new cyst formation [5,9]. This complexity underscores the need for further research to elucidate the interplay between viral and host factors [5,9].

The association of verrucous cysts with high-risk HPV types, particularly types 16 and 18, and their potential for malignant transformation necessitate a comprehensive diagnostic approach [3,9,10]. Meticulous histological examination is crucial for identifying cytologic atypia, which is key in determining the appropriate management strategy [3,9,10]. However, histology alone may not definitively ascertain the presence of specific high-risk HPV types, making adjunctive diagnostic methods like HPV PCR testing essential [3,9,10]. This combined diagnostic strategy ensures a more accurate diagnosis and aids in a more informed risk assessment of malignancy [3,9,10]. Although the risk may be low, this thorough approach is particularly vital in cases where the cysts exhibit atypical features, guiding clinicians toward a more cautious and tailored management plan [3,9,10].

The primary treatment for verrucous cysts is surgical excision [11]. However, the potential for malignant transformation, as indicated by histological findings, may necessitate additional management strategies [11]. These could include more extensive surgical procedures, adjunctive therapies, and long-term surveillance to monitor for recurrence or malignant progression [11]. The rarity of verrucous cysts means that standardized treatment protocols are not well-established, and management often relies on individual case assessment [11].

## Conclusions

The case demonstrates the challenge of distinguishing verrucous cysts from epidermal inclusion cysts without detailed histopathological analysis and HPV PCR testing. This research emphasizes the importance of vigilant histological examination due to the risk of malignant transformation in cysts infected with certain high-risk HPV strains. The primary treatment is surgical excision, with additional interventions considered if malignancy is detected. This case contributes to the sparse literature on verrucous cysts, highlighting their relevance in the differential diagnosis of subcutaneous nodules in adult patients.
